# Supplementary figures and images for: Cap-independent co-expression of dsRNA-sensing and NF-κB pathway inhibitors enables controllable self-amplifying RNA expression with reduced immunotoxicity
Source: eLife. 2025 Aug 29;14:RP105978. doi: 10.7554/eLife.105978 (PMC12396818; doi:10.7554/eLife.105978)

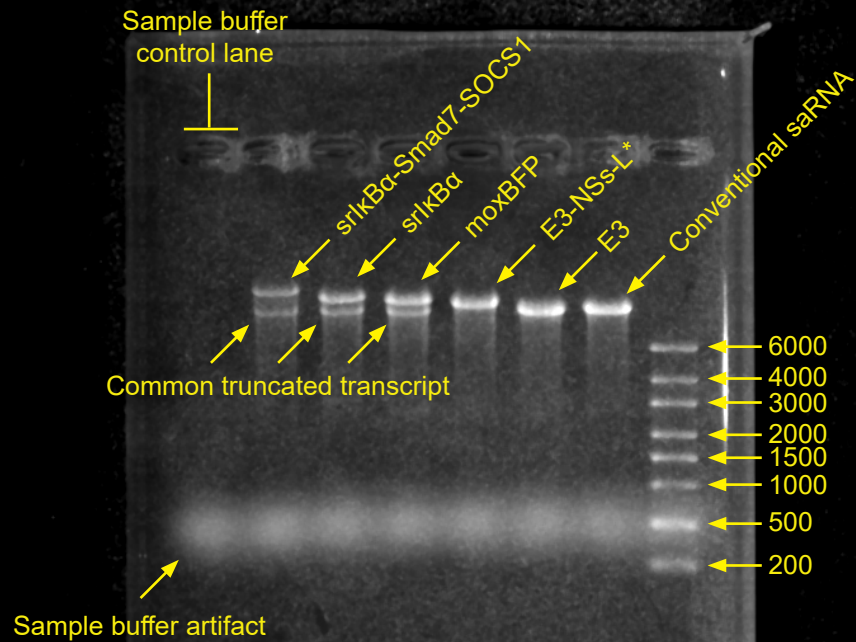

Supplement: Figure 4—figure supplement 3—source data 2. [file elife-105978-fig4-figsupp3-data2.pdf]
