# Supplementary material for: Cap-independent co-expression of dsRNA-sensing and NF-κB pathway inhibitors enables controllable self-amplifying RNA expression with reduced immunotoxicity
Source: eLife. 2025 Aug 29;14:RP105978. doi: 10.7554/eLife.105978 (PMC12396818; doi:10.7554/eLife.105978)
Supplement: MDAR checklist [file elife-105978-mdarchecklist1.docx]

**Materials Design Analysis Reporting (MDAR)**

**Checklist for Authors**

The MDAR framework establishes a minimum set of requirements in transparent reporting applicable to studies in the life sciences (see Statement of Task: doi:10.31222/osf.io/9sm4x.). The MDAR checklist is a tool for authors, editors, and others seeking to adopt the MDAR framework for transparent reporting in manuscripts and other outputs. Please refer to the MDAR Elaboration Document for additional context for the MDAR framework.

**For all that apply, please note where in the manuscript the required information is provided.**

**Materials:**

| **Newly created materials** | **indicate where provided: page no/section/legend)** | **n/a** |
| --- | --- | --- |
| The manuscript includes a dedicated "materials availability statement" providing transparent disclosure about availability of newly created materials including details on how materials can be accessed and describing any restrictions on access. | The materials availability statement (Page 45) states that all key plasmids generated in this study have been deposited with Addgene. |  |
|  |  |  |
| **Antibodies** | **indicate where provided: page no/section/legend)** | **n/a** |
| For commercial reagents, provide supplier name, catalogue number and [RRID](https://scicrunch.org/resources), if available. | Supplier names and catalog numbers of commercial antibodies can be found in the methods (page 41, 43–44) and in the key resource table (page 36-38). |  |
|  |  |  |
| **DNA and RNA sequences** | **indicate where provided: page no/section/legend)** | **n/a** |
| **Short novel DNA or RNA including primers, probes:** Sequences should be included or deposited in a public repository. | The data availability statement (page 45) states that the sequences of the plasmid constructs are provided in the source data files. |  |
|  |  |  |
| **Cell materials** | **indicate where provided: page no/section/legend** | **n/a** |
| **Cell lines:** Provide species information, strain. Provide accession number in repository **OR** supplier name, catalog number, clone number, **OR** RRID. | Supplier names and catalog numbers of the tSA201 cells can be found in the methods (page 41) and also in the key resource table (page 35). |  |
| **Primary cultures:** Provide species, strain, sex of origin, genetic modification status. | The methods section states this information, ie. *Mus musculus*, C57BL/6J, male and female, wildtype (page 40). |  |
|  |  |  |
| **Experimental animals** | **indicate where provided: page no/section/legend)** | **n/a** |
| **Laboratory animals or Model organisms:** Provide species, strain, sex, age, genetic modification status. Provide accession number in repository **OR** supplier name, catalog number, clone number, **OR** RRID. | The methods section states this information, ie. *Mus musculus*, C57BL/6J, male and female, wildtype (page 40). |  |
| **Animal observed in or captured from the field:** Provide species, sex, and age where possible. |  | X |
|  |  |  |
| **Plants and microbes** | **indicate where provided: page no/section/legend)** | **n/a** |
| **Plants:** provide species and strain, ecotype and cultivar where relevant, unique accession number if available, and source (including location for collected wild specimens). |  | X |
| **Microbes:** provide species and strain, unique accession number if available, and source. |  | X |
|  |  |  |
| **Human research participants** | **indicate where provided: page no/section/legend) or state if these demographics were not collected** | **n/a** |
| If collected and within the bounds of privacy constraints report on age, sex and gender or ethnicity for all study participants. |  | X |

**Design:**

| **Study protocol** | **indicate where provided: page no/section/legend)** | **n/a** |
| --- | --- | --- |
| If study protocol has been pre-registered, provide DOI. For clinical trials, provide the trial registration number **OR** cite DOI. |  | X |
|  |  |  |
| **Laboratory protocol** | **indicate where provided: page no/section/legend)** | **n/a** |
| Provide DOI **OR** other citation details if detailed step-by-step protocols are available. |  | X |
|  |  |  |
| **Experimental study design (statistics details)** | | |
| **For in vivo studies:** State whether and how the following have been done | **indicate where provided: page no/section/legend. If it could have been done, but was not, write not done** | **n/a** |
| Sample size determination | Not done |  |
| Randomisation | Not done, though positional balancing was occasionally performed |  |
| Blinding | Not done |  |
| Inclusion/exclusion criteria | Methods state inclusion criteria, ie. 5–12-week mice of either age, FLS utilized between passages 3 and 8 (page 40). |  |
|  |  |  |
| **Sample definition and in-laboratory replication** | **indicate where provided: page no/section/legend** | **n/a** |
| State number of times the experiment was replicated in laboratory. | All figures state the number of biological replicates. |  |
| Define whether data describe technical or biological replicates. | When technical replicates were performed, it was stated in the methods. All figures state the number of biological replicates. |  |
|  |  |  |
| **Ethics** | **indicate where provided: page no/section/legend** | **n/a** |
| **Studies involving human participants:** State details of authority granting ethics approval (IRB or equivalent committee(s), provide reference number for approval. |  | X |
| **Studies involving experimental animals:** State details of authority granting ethics approval (IRB or equivalent committee(s), provide reference number for approval. | The methods section states that mice tissue collection was conducted in accordance with the Animals (Scientific Procedures) Act 1986 Amendment Regulations 2012 and UK Home Office Project License PP5814995, with approval from the University of Cambridge Animal Welfare Review Body (page 40). |  |
| **Studies involving specimen and field samples:** State if relevant permits obtained, provide details of authority approving study; if none were required, explain why. |  | X |
|  |  |  |
| **Dual Use Research of Concern (DURC)** | **indicate where provided: page no/section/legend** | **n/a** |
| If study is subject to dual use research of concern regulations, state the authority granting approval and reference number for the regulatory approval. |  | X |

**Analysis:**

| **Attrition** | **indicate where provided: page no/section/legend** | **n/a** |
| --- | --- | --- |
| Describe whether exclusion criteria were preestablished. Report if sample or data points were omitted from analysis. If yes report if this was due to attrition or intentional exclusion and provide justification. | A single data point was excluded due to a pipetting error, as stated in the methods (ML336 concentration response experiment, page 44). qPCR data was excluded if transcripts were not detected in all samples (page 45), as there were only 3 biological replicates. |  |
|  |  |  |
| **Statistics** | **indicate where provided: page no/section/legend** | **n/a** |
| Describe statistical tests used and justify choice of tests. | All figures describe the statistical test used. All experiments performed used a repeated measures design, and thus repeated measures ANOVA (one- and two-factor) or paired t-tests were employed for statistical analysis. |  |
|  |  |  |
| **Data availability** | **indicate where provided: page no/section/legend** | **n/a** |
| For newly created and reused datasets, the manuscript includes a data availability statement that provides details for access or notes restrictions on access. | The data availability statement states that all data is available on the Figshare repository (page 45). |  |
| If newly created datasets are publicly available, provide accession number in repository **OR** DOI **OR** URL and licensing details where available. | Provided in the data availability statement (page 45): http://doi.org/10.6084/m9.figshare.27091972 |  |
| If reused data is publicly available provide accession number in repository **OR** DOI **OR** URL, **OR** citation. |  | X |
|  |  |  |
| **Code availability** | **indicate where provided: page no/section/legend** | **n/a** |
| For all newly generated custom computer code/software/mathematical algorithm or re-used code essential for replicating the main findings of the study, the manuscript includes a data availability statement that provides details for access or notes restrictions. | The data availability statement states that the unmixing analysis script is available on github (page 45). |  |
| If newly generated code is publicly available, provide accession number in repository, **OR** DOI **OR** URL and licensing details where available. State any restrictions on code availability or accessibility. | URL: https://github.com/lariferg/spectral_unmixing (page 45) |  |
| If reused code is publicly available provide accession number in repository **OR** DOI **OR** URL, **OR** citation. |  | X |

**Reporting**

MDAR framework recommends adoption of discipline-specific guidelines, established and endorsed through community initiatives. Journals have their own policy about requiring specific guidelines and recommendations to complement MDAR.

| **Adherence to community standards** | **indicate where provided: page no/section/legend** | **n/a** |
| --- | --- | --- |
| State if relevant guidelines (e.g., ICMJE, MIBBI, ARRIVE) have been followed, and whether a checklist (e.g., CONSORT, PRISMA, ARRIVE) is provided with the manuscript. | tSA201 cells were obtained from ECACC, and confirmed negative for mycoplasma as stated in methods (Page 41). |  |
